# Rapid Detection of *Mycoplasma pneumoniae* by Loop-Mediated Isothermal Amplification (LAMP) in Clinical Respiratory Specimens

**Published:** 2019-05

**Authors:** Maryam ARFAATABAR, Narjes NOORI GOODARZI, Davoud AFSHAR, Hamed MEMARIANI, Ghasem AZIMI, Ensieh MASOORIAN, Mohammad Reza POURMAND

**Affiliations:** 1. Department of Pathobiology, School of Public Health, Tehran University of Medical Sciences, Tehran, Iran; 2. Department of Microbiology and Virology, School of Medicine, Zanjan University of Medical Sciences, Zanjan, Iran; 3. Biotechnology Research Center, Pasteur Institute of Iran, Tehran, Iran; 4. Department of Internal Medicine, Shahed University of Medical Sciences, Tehran, Iran; 5. Biotechnology Research Center, Tehran University of Medical Sciences, Tehran, Iran

**Keywords:** *Mycoplasma pneumoniae*, PCR, Loop-mediated isothermal amplification (LAMP), Culture

## Abstract

**Background:**

*Mycoplasma pneumoniae* is a common cause of community-acquired pneumonia (CAP) worldwide, especially among children and debilitated populations. The present study aimed to investigate a loop-mediated isothermal amplification (LAMP) technique for rapid detection of *M. pneumonia*e in clinical specimens collected from patients with pneumonia.

**Methods:**

Throat swabs were collected from 110 outpatients who suffered from pneumonia. Throat swab samples were obtained from patients referred to the hospital outpatient clinics of Tehran University hospitals, Iran in 2017. The presence of *M. pneumonia*e in the clinical specimens was evaluated by LAMP, PCR and culture methods. Sensitivity and specificity of the LAMP and PCR assays were also determined.

**Results:**

Out of 110 specimens, LAMP assay detected *M. pneumoniae* in 35 specimens. Detection limit of the LAMP assay was determined to be 33fg /μL or ∼ 40 genome copies/reaction. Moreover, no cross-reaction with genomic DNA from other bacteria was observed. Only 25 specimens were positive by the culture method. The congruence between LAMP assay and culture method was ‘substantial’ (ϰ=0.77). Specificity and sensitivity of LAMP assay were 88.2%, 100% in compare with culture. However, the congruence between LAMP assay and PCR assay was ‘almost perfect’ (ϰ=0.86). Specificity and sensitivity of LAMP assay were 92.5%, 100% in compare with PCR.

**Conclusion:**

Overall, the LAMP assay is a rapid and cost-efficient laboratory test in comparison to other methods including PCR and culture. Therefore, the LAMP method can be applied in identification of *M. pneumoniae* isolates in respiratory specimens.

## Introduction

*Mycoplasma pneumoniae* has been recognized as an important cause of respiratory tract infections, including community-acquired pneumonia (CAP) in children and young adults (1, 2). According to Centre of Disease Control (CDC), 1 to 10 out of every 50 cases of CAP are caused by *M. pneumoniae* (3). *M. pneumoniae* accounts for up to 40% of CAP in children admitted to the hospitals (4). In addition, the organism is responsible for extra-pulmonary manifestations, including neurological diseases, hemolytic anemia, polyarthritis and erythema multiform (5). β-lactam antibiotics used for empirical therapy of respiratory tract infections are ineffective against *M. pneumoniae*, so early detection of the pathogen can be useful for commencement of appropriate antimicrobial therapy (2). Therefore, rapid and effective detection of *M. pneumoniae* may decrease length of hospital stay, emergence of antibiotic resistance and overall cost of health care (6).

Although several assays are available for diagnosis of *M. pneumoniae* infections including culture, serology and nucleic acid amplification techniques, they do not have appropriate specificity and sensitivity (7). For instance, bacterial culture is not only time-consuming but also relatively insensitive (2). Serological tests require acute-phase and convalescent-phase serum specimens and thus allowing only a retrospective diagnosis (7). The nucleic acid amplification methods such as polymerase chain reaction (PCR) have been employed, enabling fast and sensitive detection of bacteria (6). However, PCR-based methods require expensive and complicated laboratory equipment along with technical expertise, which are not readily available, particularly in the developing countries (8). In line with above-mentioned reasons, there is need for discovery and testing the applicability of novel diagnostic methods.

A powerful innovative nucleic acid amplification technique called loop-mediated isothermal amplification (LAMP) was provided highly efficient results (9, 10). This technique has some advantages when compared with other molecular techniques, such as PCR (11). DNA synthesis using Bst DNA polymerase with auto cycling strand displacement activity allows for rapid amplification of the target DNA without initial denaturation (12). Rapid amplification of target sequence performed in isothermal conditions (60 to 65°C), thereby obviating the requirement for sophisticated and expensive equipment such as thermocycler (13). LAMP reaction had higher tolerance for inhibitors present in the clinical samples (14). Furthermore, the results can be monitored in real-time through measurement of either turbidity or fluorescence, further making it possible to detect amplification without the need for the usual DNA extraction, wash steps and gel electrophoresis (15).

At present, several methods such as culture, enzyme-linked immunosorbent assay (ELISA), and PCR are usually used for the detection of *M. pneumoniae* in our region (16–18). In another study, the *M. pneumoniae* isolates were identified by PCR assay compared with culture (19). We have assessed the sensitivity and specificity of this assay in comparison to a culture and PCR methods. To the best of our knowledge, there is a paucity of data concerning the detection of *M. pneumoniae* strains via LAMP method in Iran, prompting us to investigate a LAMP assay for rapid detection of *M. pneumoniae* in clinical specimens in Tehran, Iran.

## Methods

### Clinical specimens

Over a period of 5 months between Jan and June 2017, throat swab samples were obtained from 110 outpatients with symptoms of acute lower respiratory tract infection who had been to the hospital outpatient clinics of Tehran University hospitals, Tehran, Iran.

The diagnoses were confirmed by clinical symptoms including fever, chill, cough, presence or absence of productive sputum, chest pain, abnormal breathing sounds and radiographic pulmonary abnormalities that were at least segmental. In addition, throat swab specimens collected from 30 healthy candidates were used as negative controls.

The protocol of study was reviewed and approved by the Ethics Committee of Tehran University of Medical Sciences.

### Design of LAMP primers

A set of four primers including two outer primers (F3 and B3) and two inner primers (Forward inner primer and backward inner primer) were designed using PrimerExplorerV5 software targeting the *gyrB* gene (GenBank accession no. M21519.1) (Table 1).

**Table 1:** Sequences of LAMP primers within *gyrB* gene used in the current study

***Primer***	***Length (bp)***	***Sequence (5′ to 3′)***
F3	21	CTTAAAATCCAACACAGCCTT
B3	23	CAATGAAATTAAATGGATCGGTT
FIP	50	ACCCCGTTGAATTTTCTAGTGGATTAACTAAAGGACTTAAAAAGATTGCC
BIP	46	AACGGTTCCAATGGTAAACAAATCTTGATTTCATAAACAGAGAGCT

### Genomic DNA Extraction

DNA was extracted from bacterial strains within throat swab samples using a tissue Genomic DNA Extraction Kit (Yekta Tajhiz, Tehran, Iran) according to manufacturer’s instruction and stored at −20 °C until use.

### LAMP reaction

Several reactions were applied to optimize LAMP reaction temperature. Accordingly, the best temperature for the LAMP assay was set as 62 °C and then maintained for all subsequent amplifications. Amplification was performed using a thermoblock (9). The LAMP reactions was performed in 25 μL total volume containing 2.5 μL *Bst* DNA polymerase buffer, 1.6 μM of each FIP and BIP primers, 0.2 μM of each F3 and B3 primers, 1.4 mM of each deoxynucleotide triphosphate (dNTP), 0.8 M betaine, 8 units of *Bst* DNA polymerase (New England BioLabs, Ipswich, MA) and 2 μL of DNA. The mixture was incubated at 62 °C for 60 min in a thermoblock (Dena gene Tajhiz, Iran)

### Analysis of LAMP products

In order to purify the LAMP products from positive reactions, a Gel / PCR Purification kit (Yekta Tajhiz, Tehran, Iran) was used according to the manufacturer’s instructions. Purified amplicons were digested with 8 units of the restriction endonuclease *AluI* (New England BioLabs, Ipswich, MA, United States) at 37 °C for 60 min. Purified amplicons and digested products were electrophoresed on 3% gel agarose, stained with FluoroDye DNA Fluorescent Loading Dye 10000X (SMOBIO, Hsinchu, Taiwan) and finally visualized using a GelDoc ^TM^ EZ Imager (Bio-Rad Laboratories, Inc., California, United States). In addition, the LAMP products were also sequenced to validate the accuracy of the LAMP assay.

### Sensitivity and specificity of the LAMP primers

The analytical sensitivity of the LAMP assay was determined by serial 10-fold dilutions of *M. pneumoniae* M129 genomic DNA. The same dilutions were evaluated by PCR in order to compare the detection limit of the LAMP assay with conventional PCR assay. Sterile double-distilled water (ddH_2_O) and 10 ng/μL DNA were used as negative and positive controls, respectively. Furthermore, total DNA extracted from a panel of 20 different bacterial strains (Table 2) was tested with LAMP assay at a concentration of 2 ng/μL in order to assess the potential cross-reactivity of the *M. pneumoniae* LAMP primers (6).

**Table 2:** Bacterial species tested to evaluate specificity of *M. pneumoniae* LAMP assay

***Strain and category***
Mycoplasmataceae family members
*Mycoplasma hominis* (Isolate)
*Mycoplasma pneumoniae* (ATCC 29342)
*Mycoplasma gallisepticum* (ATCC 19610)
*Mycoplasma synoviae* (ATCC 25204)
*Ureaplasma urealyticum* (Isolate)
Non-Mycoplasma species
*Escherichia coli* (ATCC 25922)
*Klebsiella pneumoniae* (ATCC 700603)
*Neisseria meningitides* (ATCC 13077)
*Pseudomonas aeruginosa* (ATCC 10662)
*Acinetobacter baumannii* (ATCC BAA-747)
*Staphylococcus aureus* (ATCC 25923)
*Staphylococcus epidermidis* (ATCC 14990)
*Streptococcus agalactiae* (Isolate)
*Streptococcus pneumoniae* (Isolate)
*Streptococcus pyogenes* (ATCC 19615)
*Enterococcus faecium* (ATCC 51299)
*Enterococcus faecalis* (ATCC 29212)
*Nocardia asteroids* (Isolate)
*Nocardia farcinica* (Isolate)

### PCR assay

PCR assays targeting *p1* gene were performed using specific primers as previously described (16). The PCR amplicons were electrophoresed in 1% gel agarose and stained with FluoroDye DNA.

### Culture

Swab specimens were cultured in PPLO Broth medium and incubated at 37 °C in 5% carbon dioxide up to 20 d (20). The media plates were then examined for the presence of fried-egg-shaped colonies which are characteristic of the colonial growth of Mycoplasmas.

## Results

There were 43 males with the mean ages of 54±18 yr and 67 females with the mean ages of 56±18 yr. The underlying diseases included diabetes mellitus, cardiovascular disease, and kidney failure among 16, 24, and 5 patients respectively. The major and minor clinical signs of the patients were dyspnea (67.3%) and fever (14.5%). Out of 110 outpatients, LAMP assay detected *M. pneumoniae* in 35 cases. Of these 35 (31.8%) cases, 20 (29.9%) were female and the remaining 15 (34.9%) were males. The highest number of positive cases occurred in age group of >60 yr (*n*=14, 40%), whereas the lowest numbers were observed in age group of 40–60 yr (*n*=12, 25%) (Table 3).

**Table 3:** Relationship between clinical features by age and gender of the patients with the outcome of LAMP assay in patients with atypical pneumonia

***Variable***		***M. pneumonia***			***P-value***	***Odds ratio (OR)***	***95% Confidence Interval***
		***Positive No (%)***	***Negative No (%)***	***Total (%)***			
Age(yr)							
	1–20	5(41.7)	7(58.3)	12(10.9)	0. 87	1.13	(0.24–5.30)
	20–40	4 (26.7)	11(73.3)	15(13.6)	0.08	0.22	(0.04–1.25
	40–60	12(25)	36(75)	48(43.6)	0.17	0.44	(0.13–1.42)
Sex	Male	15(34.9)	28(65.1)	43(39.1)	0.18	1.96	(0.72–5.35)
	Female	20(29.9)	47(70.1)	67(60.9)			
Chest Pain	Yes	21(39.6)	32(60.4)	53(48.1)	0.004	4.96	(1.66–14.7)
	NO	14(24.6)	43(75.4)	57(51.8)			
Fever	Yes	3 (18.8)	13(81.3)	16(14.5)	0.11	0.24	(0.04–1.38)
	NO	32(34)	75(68.2)	107(97.2)			
Lethargy	Yes	16(29.6)	38(70.4)	54(49.1)	0.40	1.62	(0.51–5.09)
	NO	19(33.9)	37(66.1)	56(50.9)			
Headache	Yes	6(18.8)	26(81.3)	32(29.1)	0.01	0.17	(0.04–0.70)
	NO	29(37.2)	49(62.8)	78(70.9)			
Sore Throat	Yes	8(25.8)	23(74.2)	31(64.5)	0.22	2.26	(0.60–8.39)
	NO	27(34.2)	52(65.8)	39(35.5)			
Cough	Yes	23(32.4)	48(67.6)	71(64.5)	0.71	1.25	(0.38–4.06)
	NO	12(30.8)	27(69.2)	39(35.5)			
Sputum production	Yes	12(27.9)	31(72.1)	43(39.1)	0.40	0.59	(0.17–2.00)
	NO	23(34.3)	44(65.7)	67(60.9)			
Dyspnea	Yes	21(28.4)	53(71.6)	74(67.3)	0.08	0.37	(0.12–1.12)
	NO	14(38.9)	22(61.1)	36(32.7)			
Anxiety	Yes	6(23.1)	20(76.9)	26(23.6)	0.72	0.77	(0.18–3.21)
	NO	29(34.5)	55(65.5)	84(76.4)			
Diabetes	Yes	7(43.8)	9(56.3)	16(14.5)	0.99	0.99	(0.25–3.86)
	NO	28(29.8)	66(70.2)	94(85.5)			
Cardiovascular disease	Yes	8(33.3)	16(66.7)	24(21.8)	0.97	1.02	(0.31–3.31)
	NO	27(31.4)	59(68.6)	86(78.2)			
Kidney Failure	Yes	3(60)	2(40)	5(4.5)	0.11	6.86	(0.64–73.43)
	NO	32(30.5)	73(69.5)	105(95.5)			

**Fig. 1:**
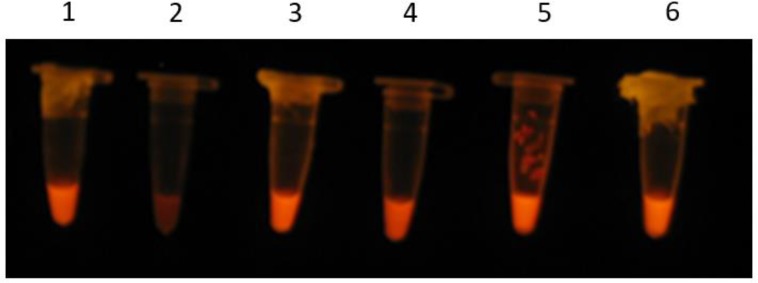
Detection of LAMP product with GelRed ™ stain under UV light. Lane 1: Positive control (M129 strain). Lane 2: Negative control, reaction mixture without DNA template. Lane 3–6: Clinical isolets positive by LAMP assay

**Fig. 2:**
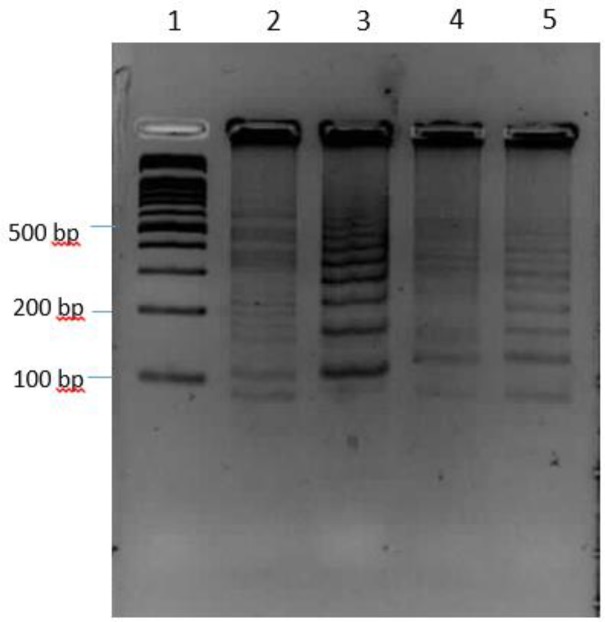
Electrophoresis of LAMP products in 3% agarose gel with FluoroDye DNA Fluoresent Loading Dye 10000X stain. Lane 1: 100 bp DNA marker, Lane 2–5: LAMP product of clinical isolates

**Fig. 3:**
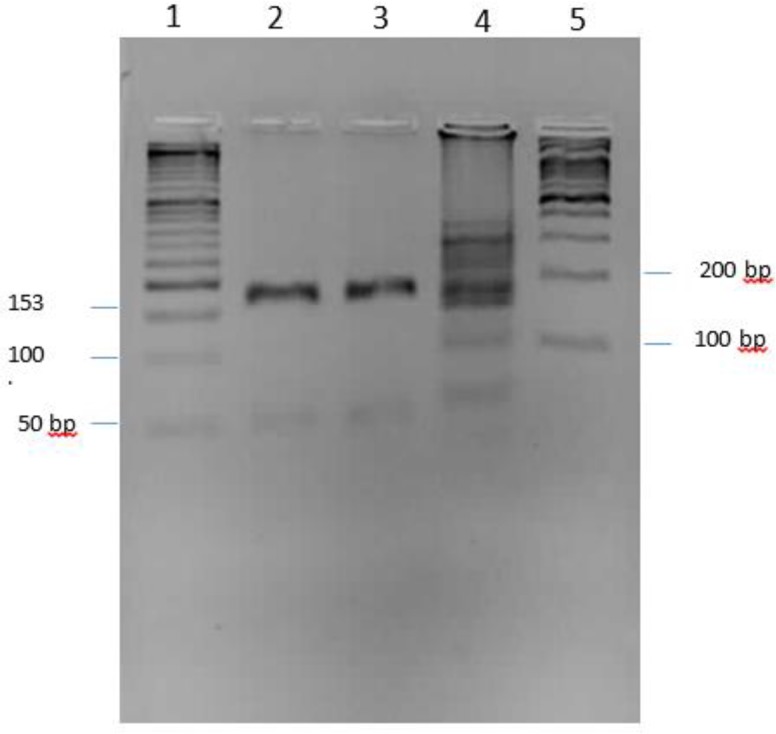
Aarose gel electrophoresis of LAMP products after digestion with the *AluI* restriction enzyme. Lane 1: 50 bp DNA marker. Lane 2: digested product of M129 strain. Lane 3: digested products of clinical isolates, Lane 4: undigested LAMP product of M129 strain. Lane 5: 100 bp DNA marker

As for analysis of LAMP reactions, the products were shown by direct visualization of a color change after staining (Fig. 1). The amplification was produced as a ladder-like pattern on the gel electrophoresis (Fig. 2). The accuracy of reactions was also confirmed by digestion with *AluI*. The digested fragments were 153 bp and 49bp in sizes, which is in accordance with the predicted sizes (Fig. 3).

**Fig. 4:**
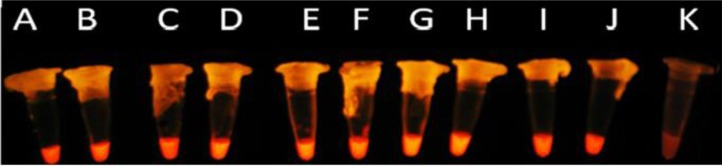
The detection limit of the LAMP assay was determined by serial 10-fold dilutions of *M. pneumoniae* M129 genomic DNA; Columns A (3.3 ng/μL) to J (33 fg/μL). K; Control Negative (reaction mixture without DNA template).

The sensitivity of LAMP assay was determined by testing 10-fold serial dilutions of *M. pneumoniae* M129 genomic DNA and detection limit obtained to be 3.3×10^−8^ μg/μL, or 3.74 ×10^1^ cfu/μL. Furthermore, absence of cross-reaction with genomic DNA from other bacteria indicated the high specificity of LAMP assay (Fig. 4).

The effectiveness of LAMP assay was also compared with that of PCR and traditional culture methods. Out of the 110 respiratory specimens, 35 (31.4%) were positive by the LAMP assay, 29 (26.3%) were positive by PCR and 25 (22.7%) were positive by culture method. Our study also showed that LAMP assay is more sensitive than the culture method (**P**=0.002). LAMP was also shown to be superior to PCR in terms of detecting *M. pneumoniae* in clinical specimens (*P*=0.031). Comparison of LAMP assay results with those of other methods are shown in (Table 4). The LAMP assay was shown to have positive and negative predictive values of 71.4% and 100%, respectively.

**Table 4:** Comparison of the LAMP assay results with Culture and PCR methods

	***LAMP +***	***LAMP −***	***Se %***	***Sp %***	***PPV (%)***	***NPV (%)***	***P-value***	***ϰ-value***
Culture+	25	0	100	88.2	71.4	100	0.002*	0.77
Culture −	10	75						
PCR+	29	**0**	100	92.5	82.5	100	0.031	0.86
**PCR–**	6	75						

Se; Sensitivity, Sp; Specificity, PPV; Positive predictive value, NPV; Negative predictive value, Kappa statistic and strength of agreement: <0.00; Poor, 0.00–0.20; Slight, 0.21–0.40; Fair, 0.41–0.60; Moderate, 0.61–0.80; Substantial, 0.81–1.00; Almost Perfect

*P*-values less than 0.05 are statistically significant

## Discussion

As a promising diagnostic tool, LAMP assay enables rapid specific detection of different pathogens in humans and animals under isothermal conditions, which is readily available for use in low-income countries where there is insufficient infrastructure (21).

In the present study, the LAMP assay could be useful in detection of *M. pneumoniae* in clinical specimens of patients with CAP. Currently, culture and serological assays are still considered as standard laboratory methods for the specific identification of *M. pneumoniae* infections (2, 22, 23). However, the LAMP assay is superior to above-mentioned conventional methods, which is often time-consuming (2, 8, 23, 24).

Several LAMP procedures targeting *23S rRNA* (26), *SDC1* sequence (2), *P1* gene (7), and CARDS toxin gene (6) have been successfully developed for detection of *M. pneumoniae* in clinical specimens. Moreover, the technique has been commercialized for diagnostic testing in the USA and Japan (25). We targeted *gyrB* for LAMP assay because it has repeated sequences and is highly conserved among *M. pneumoniae* species. The designated primers are specific to *M. pneumoniae* and don’t have any cross-reactivity with other bacterial species.

The LAMP assay is more sensitive and reliable than conventional culture and serology methods for detection of *M. pneumoniae*. In a cross-sectional study, the LAMP method was compared with culture and serology. The LAMP assay had a significant sensitivity of 96.8% and specificity of 100%, whereas this value for EIA and the particle agglutination tests, were 38.7%, 19.4%, and 76.9% and 93.1%, respectively (24). A sensitivity of 100% and the specificity of 99% were reported by LAMP assay in comparison with culture from *M. pneumoniae* reference strains and clinical isolates (8). Another study in the United States compared the LAMP and real-time PCR assays for detection of *M. pneumoniae* from several respiratory specimens. The sensitivity and specificity of the LAMP assay were observed to be 88.5% and 82.1%, respectively (6). The LAMP assay was compared with PCR using clinical specimens for the detection of *M. pneumoniae* in children. The LAMP assay had 100% sensitivity and specificity compared with the PCR method (26). Our study showed sensitivity and specificity of 100% and 92.5% LAMP assay in comparing PCR that close to the reported studies. In some studies, reported detecting *M. pneumoniae* by LAMP assay at concentrations as low as 10 fg/ μL, or 11 genome copies (6). These reports are similar to our finding, LAMP assay was able to detect up to 33 fg /μL of genomic DNA (7). The characteristics of *M. pneumoniae* such as lack of a cell wall, fragility during sample collection or transport, inherent slow growth in culture makes it a perfect candidate for this promising approach (6). In general, simplicity, ruggedness, and cheapness of LAMP assay could provide major advantages over traditional techniques (1, 21).

## Conclusion

LAMP assay is more specific for the detection of *M. pneumoniae* with high efficiency. The technique is a rapid and cost-efficient laboratory test compared to other methods including PCR and culture methods.

## Ethical considerations

Ethical issues (Including plagiarism, informed consent, misconduct, data fabrication and/or falsifications, double publication and/or submission, redundancy, etc.) have been duly considered by the authors.
